# Impact of Allele-Specific Expression on Ripening and Quality Characteristics of ABB Banana Fruit

**DOI:** 10.3390/ijms26094090

**Published:** 2025-04-25

**Authors:** Fang Fang, Bin Liu, Qiuzi Chen, Lisi Xiao, Zhuozi Deng, Zhaoqi Zhang, Xuemei Huang, Xuequn Pang

**Affiliations:** 1 College of Horticulture, South China Agricultural University, Guangzhou 510642, China; fangfang@scau.edu.cn (F.F.); liub@scau.edu.cn (B.L.); 13719947978@163.com (Q.C.); dengzhuozi@126.com (Z.D.); zqzhang@scau.edu.cn (Z.Z.); 2 State Key Laboratory for Conservation and Utilization of Subtropical Agro-Bioresources/Guangdong Provincial Key Laboratory of Postharvest Science of Fruit and Vegetables/Engineering Research Center for Postharvest Technology of Horticultural Crops in South China, South China Agricultural University, Guangzhou 510642, China; lisi.xiao@oebiotech.com; 3 College of Life Sciences, South China Agricultural University, Guangzhou 510642, China

**Keywords:** banana, allele-specific expression (ASE), transcriptome, gene expression, ethylene

## Abstract

Allele-specific expression (ASE) is a phenomenon in which the expression level of an allele from both parents is inconsistent, which is considered to play a key role in the differences between hybrids. As a typical climacteric fruit, banana undergoes a ripening process that affects the quality of the fruit. BaXi (*Musa*, AAA group) and Fen Jiao (*Musa*, ABB group) banana fruits show different traits during postharvest ripening, and their high-quality reference genomic sequences have been published. In this work, we analyzed differentially expressed genes (DEGs) in these two banana cultivars based on the transcriptomes during the postharvest stages. Additionally, the imbalance expression of alleles of DEGs in Fen Jiao banana fruit was analyzed, revealing that 27.2% (3 d) and 22.2% (6 d) of the 15,415 DEGs showed ASE. Then, the ASE profiles related to the post-ripening of banana fruit were built, focusing on ripening-related pathways, such as ethylene biosynthesis (62.5–83.3%), starch degradation (0–75%) and cell wall material degradation (34.6–90.9%). The ASE genes involved in ripening were more frequent than those associated with general gene expression. In addition, the candidate key genes of ASE alleles involved in ethylene synthesis and starch degradation were identified, including the alleles of *MaACS7*/*MbACS7*, *MaACO2*/*MbACO6*, *MaACO3*/*MbACO7*, *MaACO8*/*MbACO13* and *MaACO6*/*MbACO17* involved in ethylene biosynthesis, and those of *MaAMY1*/*MbAMY3*, *MaBMY1*/*MbBMY2*, *MaBMY7*/*MbBMY8* and *MaDPE2*/*MbDPE2* involved in starch degradation. The expression of the B genes of these key enzyme genes (*ACS*/*ACO*/*AMY*) is more active than that of the A genes in Fen Jiao bananas.

## 1. Introduction

Allele-specific expression (ASE) has been referred to as an imbalance in mRNA abundance between alleles, with the paternal allele being more highly expressed than the maternal allele or vice versa. ASE happens when the alleles at a locus in an organism with diploid or higher ploidy show different states or activities. ASE plays a critical role in normal development and various cell functions, and as the number of chromosome sets increases, the complexity of ASE also increases [1].

In plants, the impact of asymmetric ASE on the phenotype of heterozygous species has been revealed, such as maize [2,3,4,5], Arabidopsis [6,7,8], barley [9], rice [10] and apple [11]. It was found that 60% of genes displayed ASE in a maize hybrid. The regulation of ASE in response to environmental stress indicates that the parental alleles have different roles in hybrids [2]. It has also been reported that 43–53% of the 316 analyzed genes showed unequal allelic expression [4]. In barley, more than 63% of the genes tested showed ASE, and the imbalance was affected by genetic variation, developmental stages and drought conditions [9]. In rice hybrids, ASE genes were significantly associated with drought-response quantitative trait loci (QTL) markers under water stress, highlighting that significant trait-related genes showed ASE in the F1 generation [10]. The ASE assay might serve as an effective method to identify candidate genes related to the phenotype of hybrids.

Bananas are one of the most important fruit crops cultivated in tropical and subtropical regions and are consumed all over the world [12]. Cultivated bananas with various genotypes are hybrids, diploids or polyploids, resulting from the crossbreeding of two wild diploid species, *Musa acuminata* (AA genotype) and *Musa barbarian* (BB genotype) [13,14]. Baxi Jiao (*Musa* spp., AAA group, Cavendish subgroup) and Fen Jiao (*Musa* spp., ABB group, Pisang Awak subgroup) are banana cultivars widely grown in China. These banana varieties differ in their postharvest performance and quality traits of fruits, with Fen Jiao fruit ripening more quickly and having a shorter shelf-life than Baxi Jiao fruit. The ripening of banana fruit is a complex process and is closely related to postharvest traits such as color, flavor and softening, which make the fruit edible and attractive for consumption. Genes related to ripening have been identified and characterized in different banana cultivars of different genotypes, and the regulation of hormones and transcription factors in fruit ripening has been intensively studied [15]. However, there is limited information regarding the fruit ripening features and formation mechanism of banana hybrids. Recent studies have provided reference genomes for these two cultivars; however, the assemblies are likely to have missed the ASE underlying important selected traits. Limited studies have been conducted on ASE in bananas and, therefore, it is still poorly understood. The postharvest banana fruit exhibits distinct stage changes during the ripening process, making it an excellent material to study ASE. Our previous study showed that the ASE of the *SGR1* allele significantly influences the different traits for fruit de-greening of banana cultivars (AAA and AAB/ABB) under high temperatures [16]. We suggested that ASE is widely present in banana fruit during ripening, which has an important impact on the formation of post-ripening characteristics and quality.

Many methods have been developed to analyze ASE, generally depending on RNA availability and genetic polymorphisms, such as single-nucleotide polymorphisms (SNPs). RNA-seq analysis of rice hybrids identified over 3000 ASE genes through parental genome comparison [17]. The development of high-throughput sequencing technology has been widely used to study ASE, providing a model for investigating allelic variation that may play an important role during evolution [18]. The banana genome sequence has been published in (https://banana-genome-hub.southgreen.fr/) (accessed on 25 April 2024). Banana is a good plant material for studying postharvest fruit ripening and quality development. High-throughput technology provides technical support for us to study the regulatory mechanism underlying the allelic preference expression in banana fruit.

This study investigated the ASE contributing to ripening features and quality variation traits in banana. The ASE genes expressed during Fen Jiao fruit ripening were identified using RNA-seq data. The important candidate ASE genes controlling for the correlated differences in ethylene biosynthesis and fruit texture were screened. The findings from this study provide a theoretical basis for the genetic improvement and quality management of banana fruit.

## 2. Results

### 2.1. Analysis of Allele-Specific Expression (ASE) of Differentially Expressed Genes (DEGs) in Fen Jiao During Fruit Ripening

An RNA-seq approach was used to investigate the ASE profiles during banana ripening. Libraries were constructed of the three ripening stages (green, turning, and full-ripening stages) of BX and FJ. The sequencing process generated 7,554,406 raw reads from the BX libraries and 7,487,784 from the FJ libraries. Principal component analysis (PCA) was performed, showing that two biological replicates from the same time point clustered closely together, whereas samples from different time points were distinctly separated (Supplementary Figure S1). All clean reads were aligned to the *Musa* reference genome (composed of A-genome and B-genome) to annotate the genes; most sequences of BX (93.24–93.63%) and FJ (96.15–96.45%) matched the reference genome (Supplementary Table S1). A total of 25,716 allele pairs were obtained in FJ (ABB). To further explore the mechanism of ASE related to the ripening process in FJ, we focused on the ASE of DEG alleles during fruit ripening. The DEG alleles were identified by screening the genes (A or B from allele genes) that were upregulated or downregulated by more than 2-fold during the ripening process of both BX and FJ banana fruits. We considered 2-fold expression ratios for DEGs in the ripening stages. In total, 15,415 DEG alleles from 25,716 A-B allele pairs were obtained in FJ.

**Figure 1:**
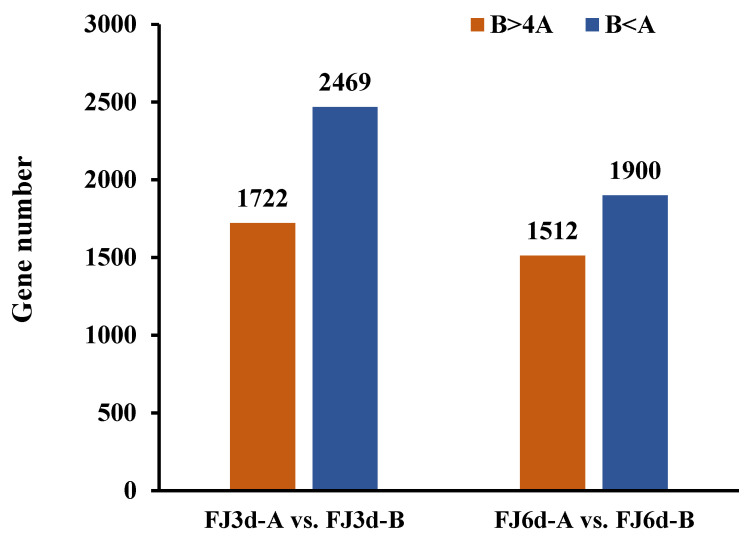
Allele-specific expression (ASE) of differentially expressed genes (DEGs) identified in FJ fruit during postharvest ripening. ASE genes were identified on the basis of fold expression ratios (B > 4A or B < A) as allelic expression imbalances; B > 4A indicates the specific expression of B allelic genes in FJ, while B < A indicates the specific expression of A allelic genes in FJ.

In normal diploid heterozygous loci (1:1), 2-fold threshold has become the standard in both plant and animal allele-specific expression (ASE) studies. Here, the allele frequency of A and B alleles in FJ banana (ABB) is 1:2, and ASE refers to the phenomenon in which the allele frequency significantly deviates from this ratio. We considered the fold expression ratios (B > 4A or B < A) as indicating ASE. Among the 15,415 DEG alleles identified from FJ fruit during postharvest ripening, 1722 B genes were upregulated (B > 4A) and 2469 B genes were downregulated (B < A) in FJ-3d fruit, while 1512 B genes were upregulated (B > 4A) and 1900 B genes were downregulated (B < A) in FJ-6 d fruit (Figure 1). Overall, 27.2% (3 d) and 22.2% (6 d) of the 15,415 DEGs showed ASE in FJ fruit. These findings indicate that ASE is widely present in DEGs during FJ fruit ripening.

### 2.2. GO and KEGG Enrichment Analyses of ASE Genes in FJ Fruit

To identify the major functional categories of the target DEGs showing ASE in FJ fruit, GO enrichment analysis was performed at two postharvest time points (3 d and 6 d) (Supplementary Figure S2). The DEGs showing ASE were organized into three major GO categories. In the biological process category, “metabolic processes” and “cellular process” exhibited the most significant differences in ASE gene expression changes during FJ fruit ripening. For the cellular component category, the majority of genes fell under the “cell” and “cell part” classifications. Meanwhile, “catalytic activity” and “binding” were the most prevalent functions in the molecular function category.

**Figure 2:**
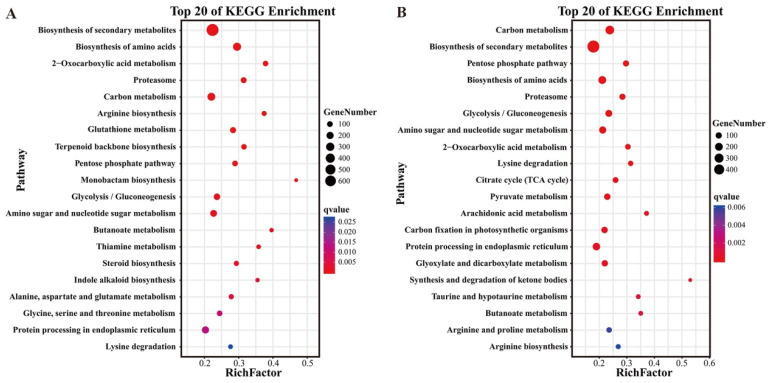
The top 20 significantly enriched KEGG pathways of DEGs, showing ASE in FJ fruit. (**A**) Enrichment analysis of KEGG pathways of DEGs at 3 DPH; (**B**) enrichment analysis of KEGG pathways of DEGs at 6 DPH. The Rich Factor represents the proportion of ASE-associated genes mapped to a given pathway relative to all annotated genes in that pathway. The q-value represents FDR-adjusted *p*-value [0, 1] (the lower it is, the more significant).

To investigate the biological functions of ASE genes, significantly enriched pathways (q-value ≤ 0.05) were identified by mapping ASE genes to the KEGG database. Figure 2 displays the top 20 enriched pathways, where lower q-values indicate higher statistical significance. These results reveal key metabolic processes involved in the postharvest biological changes in FJ banana. Among the KEGG-enriched ASE genes, the top pathways included “biosynthesis of secondary metabolites”, “carbon metabolism”, “biosynthesis of amino acids” and “proteasome”, which are important for fruit ripening.

### 2.3. ASE Genes Involved in Ethylene Biosynthesis During FJ Fruit Ripening

**Figure 3:**
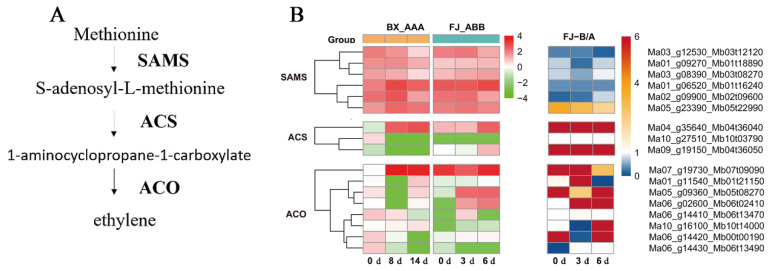
Expression pattern analysis of DEGs involved in the ethylene biosynthesis pathway that show ASE during FJ fruit ripening. (**A**) Schematic representation of the biosynthetic pathway for ethylene biosynthesis in plants. (**B**) Heatmap of the expression levels of differentially expressed ethylene biosynthesis genes (*SAMS*, *ACS* and *ACO* gene families) acquired via RNA-Seq. The rows represent genes, and the columns represent time points. We considered 2-fold expression ratios (B > 4A or B < A) as indicating allelic expression imbalances. The value of FJ-B/A is higher than 4, indicating the dominant expression of the B gene (red colors), and the value of FJ-B/A is lower than 1, indicating the dominant expression of gene A (blue colors).

The key genes involved in ethylene biosynthesis showed highly imbalanced expression levels (Figure 3 and Table 1). Within each multigene family, distinct expression patterns were observed during FJ fruit ripening, with certain members showing significant upregulation while others remained stable or were downregulated. A total of 17 DEG alleles involved in the ethylene biosynthesis pathway were identified in the FJ fruit transcriptome, including six allele pairs of S-adenosyl-L-methionine synthase (*SAMS*), three allele pairs of 1-aminocyclopropane-1-carboxylic acid synthase (*ACS*) and eight allele pairs of 1-aminocyclopropane-1-carboxylic acid oxidase (*ACO*). The transcripts of six *SAMS*, one *ACS* and two *ACO* allele pairs were upregulated in both FJ and BX banana fruits. Among these DEGs, 62.5–83.3% of the allelic genes showed imbalanced allelic expression, constituting quite a high proportion of the identified DEGs. The results suggest that ASE genes may have an important effect on ethylene synthesis during postharvest banana fruit ripening.

### 2.4. ASE Genes Associated with Starch Degradation Pathway During FJ Fruit Ripening

**Figure 4:**
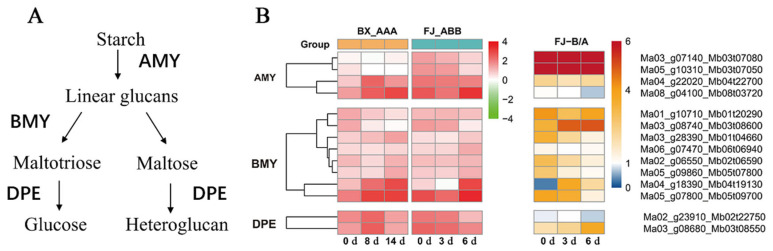
A summary of ASE genes involved in the starch degradation pathway in FJ fruit during ripening. (**A**) Schematic representation of the starch degradation pathway in plants. (**B**) Heatmap of the expression levels of *AMY*/*BMY*/*DPE* genes acquired via RNA-Seq. The ASE analysis was conducted as described in Figure 3.

Several key enzymes that mediate starch-to-sugar conversion during banana ripening have been identified, including *α*-amylases (AMYs), *β*-amylases (BMYs) and disproportionating enzymes (DPEs) [19,20]. The genes of these three families are significantly upregulated during the ripening process of FJ and BX bananas. A total of 14 differentially expressed allelic genes (4 *AMY*s, 8 *BMYs* and 2 *DPEs*) associated with the starch degradation pathway were significantly upregulated after ripening initiation. Overall, 50–75% of the *AMYs* showed unequal allelic expression, including three *AMYs*, one *BMY* and one *DPE* allelic gene. In total, 50% of *DPEs* at 6 d showed unequal allelic expression (Figure 4 and Table 2). There were more *AMY* and *BMY* genes that preferentially expressed B than those that preferentially expressed A, while for the *DPE* genes, only an A gene was found to be preferentially expressed. These results indicate that ASE may have an important effect on starch degradation during banana fruit ripening. The imbalanced expression pattern of these three gene families may be related to the faster starch degradation and greater accumulation of oligosaccharides in FJ compared to BX.

### 2.5. Ase Genes Associated with Cell Wall Degradation Pathway During FJ Fruit Ripening

**Figure 5:**
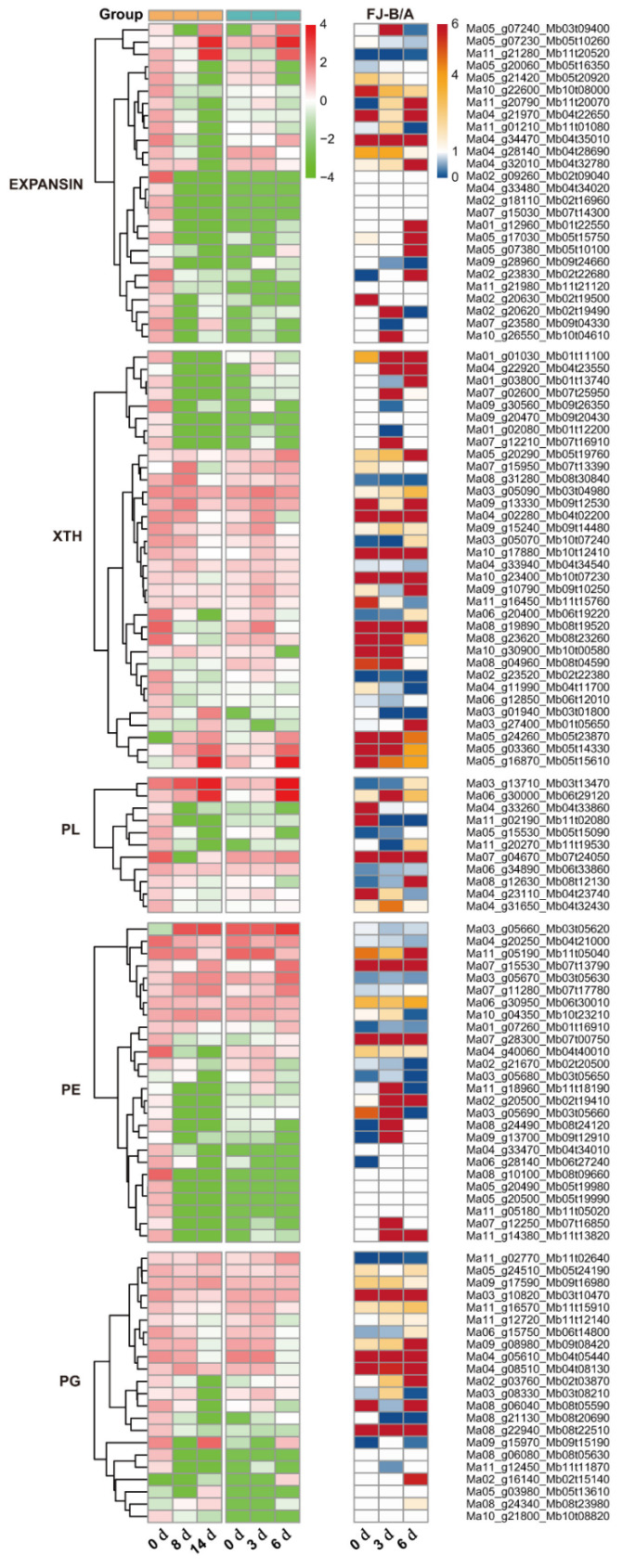
A summary of ASE genes involved in the cell wall degradation pathway in FJ fruit during ripening. The ASE analysis was conducted as described in Figure 3.

During the ripening of banana fruit, a decline in fruit firmness is largely due to the disassembly of the cell wall. Fruit softening involves coordinated action of cell wall-degrading enzymes, including polygalacturonase (PG), pectinesterase (PE), pectate lyase (PL), xyloglucan transglycosylase/hydrolase (XTH) and expansin (EXP) [21]. The GO and KEGG analyses showed that cell wall metabolism pathways were highly enriched. The DEGs including 26 *EXP*, 34 *XTH*, 11 *PL*, 26 *PE* and 22 *PG* genes were significantly upregulated or downregulated during ripening (Figure 5 and Table 3). The genes of these five families exhibited imbalanced expression during the ripening process of FJ fruit. At 3 and 6 d of the ripening stage, the *EXPANSIN*, *XTH*, *PE*, and *PG* gene families showed a large number of ASE genes, and there were more genes that specifically expressed B than those that specifically expressed A. These results demonstrate an association of ASE with the accelerated ripening and softening of bananas. Moreover, 34–90% of the DEGs showed unequal allelic expression. ASE may have an important effect on cell wall degradation during postharvest banana fruit ripening.

### 2.6. Changes in Fruit Ripening Parameters in BX and FJ Bananas

**Figure 6:**
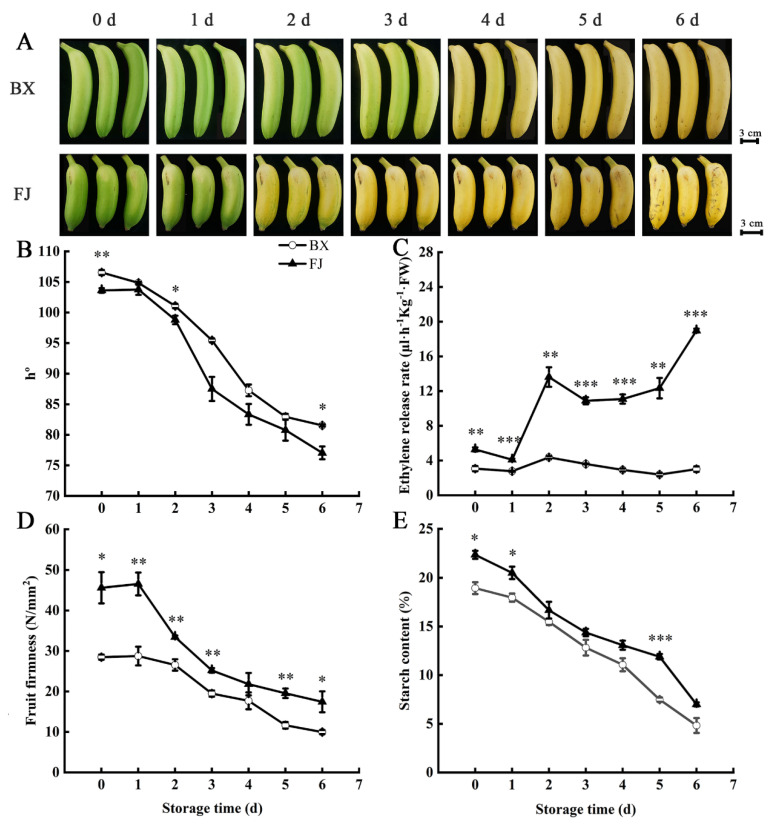
Physiological characteristics of BX and FJ banana fruits during ripening after ethylene treatment. (**A**) Photographs of BX and FJ banana fruits; scale bar = 3 cm. (**B**) Changes in fruit peel hue angle and (**C**) ethylene production in BX and FJ fruits during ripening. (**D**) Fruit firmness of BX and FJ during ripening. (**E**) Total starch levels of BX and FJ fruits during ripening. Means with error bars (SE, n = 3). Asterisks indicate significant difference between the FJ and BX fruits at each time point (*: *p* ≤ 0.05, **: *p* ≤ 0.01, and ***: *p* ≤ 0.001).

To reveal the changes in the genotypes of the BX and FJ banana cultivars during fruit ripening after ethylene treatment, important fruit ripening parameters were measured in postharvest banana fruits. The fruits of the two cultivars (BX and FJ) gradually de-greened after the ethylene treatment; the BX fruit reached the turning stage on day 3, while the FJ fruit reached that stage on day 2, and the two varieties turned completely yellow after two days later (Figure 6A). Their appearance showed that, during ripening, the process of the FJ fruit peel turning yellow was faster than that of the BX fruit, which corresponded to a decrease in the *h°* value. The FJ fruit showed a significantly faster decrease in the *h°* value than the BX fruit during storage (Figure 6B). Ethylene production in the ripening BX and FJ fruits increased significantly during storage. Ethylene production in FJ fruits exhibited a rapid and sustained increase starting at day 1, higher than BX throughout the storage (Figure 6C). The FJ fruit showed faster ripening than the BX fruit. In addition, the pulp tissue loses its turgidity and turns soft during banana fruit ripening. Fruit firmness decreased sharply on day 2 for the FJ fruit, while the firmness of the BX fruit decreased most rapidly on day 3. The FJ fruit softened completely by day 4, while for the BX fruit, it took 5 days (Figure 6D). The starch content of the FJ fruit decreased faster than that of the BX fruit. At the full-ripening stage on day 6, the starch content of the BX fruit was around 6%, while that of the FJ fruit was around 8% (Figure 6E).

### 2.7. Verification of ASE Using Real-Time qPCR

A range of genes are differentially expressed to regulate the ripening processes. The RNA-Seq revealed a great number of ASE genes related to ethylene biosynthesis, the starch degradation pathway and cell wall modification during banana fruit ripening (Figure 3, Figure 4 and Figure 5). The samples of FJ and BX fruits for RNA-seq were obtained after natural ripening and without ethylene treatment, and their gene expression was analyzed via RT-qPCR in a previous study [22]. Here, we used an ethylene treatment system to verify that these genes also exhibit ASE in the process of post-ripening. Based on an analysis of the transcriptome data, the BX fruits reached the turning and full-ripening stages without ethylene treatment at 8 and 14 DPH, respectively, whereas the FJ fruits reached the turning and full-ripening stages at 3 and 6 DPH, respectively. After ethylene treatment, sampling was conducted at 2 and 4 DPH for the FJ fruits and at 3 and 5 DPH for the BX fruits, which corresponded to the turning and full-ripening stages, respectively.

**Figure 7:**
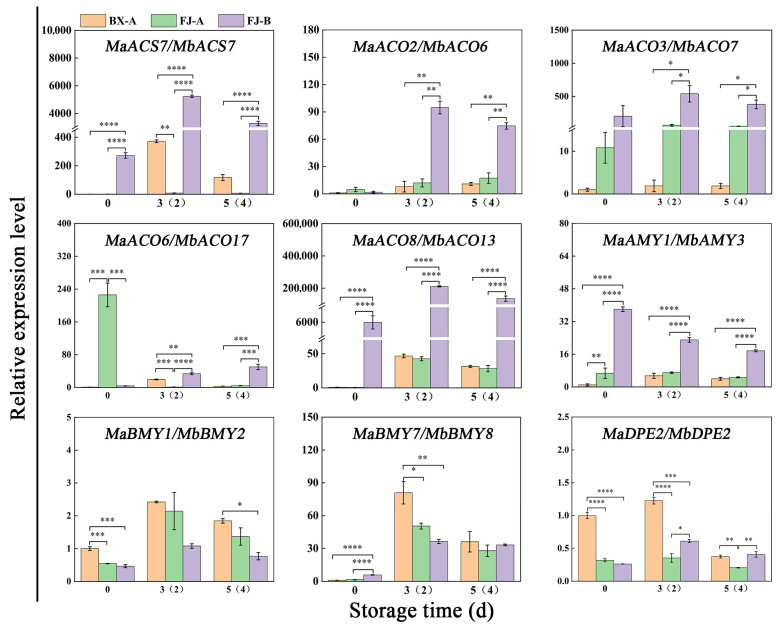
RT-qPCR validation of selected ASE genes in ethylene-treated BX and FJ fruits at three time points during storage. Expression levels (mean ± SE, n = 3) were normalized to *MaRPS4* using the 2^−∆Ct^ method, with significance levels indicated as * *p* ≤ 0.05, ** *p* ≤ 0.01, *** *p* ≤ 0.001, **** *p* ≤ 0.0001 compared with the control (BX).

The ASE genes encoding key enzymes of ethylene biosynthesis and starch degradation were screened and chosen for further RT-qPCR experiments. We performed qRT-PCR experiments to determine the expression of 1 *ACS*, 4 *ACO*, 1 *AMY*, 2 *BMY* and 1 *DPE* genes during the ripening of BX and FJ banana fruits after ethylene treatment. The RT-qPCR results are shown in Figure 7. Comparative analysis demonstrated strong concordance between RNA-seq (Supplementary Figure S3) and RT-qPCR results for the selected genes. There is an imbalance in the expression of these key genes, and the differences in these allele genes’ expression are more significant with ethylene treatment than without ethylene treatment.

The highly expressed *ACS* and *ACO* genes selected for the analysis of the banana fruit ripening process include five alleles: *MaACS7*/*MbACS7*, *MaACO2*/*MbACO6*, *MaACO3*/*MbACO7*, *MaACO8*/*MbACO13* and *MaACO6*/*MbACO17*. The expression levels of these five alleles in FJ are higher than those in BX, consistent with the higher ethylene release in FJ compared to BX. These five alleles are key genes related to the difference in ethylene synthesis rate during the post-ripening process between BX and FJ banana fruits. *MaACO2*/*MbACO6*, *MaACO3*/*MbACO7*, *MaACO8*/*MbACO13* and *MaACS7*/*MbACS7* all exhibit imbalanced expression of alleles, indicating a significant expression of the alleles in the B-genome. These ASE genes may play an important role in ethylene synthesis during FJ fruit ripening. Spearman correlation analysis demonstrated strong positive associations between ethylene evolution rates and the expression of ethylene biosynthesis ASE genes (A- and B-*ACS*/*ACO*) in FJ bananas, and the relevant A genes in BX during fruit ripening. In FJ fruits, all the selected 10 *ACS*/*ACO* ASE genes from 5 alleles, except *MaACO6*, showed significantly positive correlation with ethylene release rates. Notably, all the selected B genes showed stronger correlation than the A genes. Compared to the BX fruits, more *ACS*/*ACO* genes in FJ fruits were involved in ethylene biosynthesis (Supplementary Figure S4A,B), in agreement with the higher ethylene production in FJ than BX fruits (Figure 6C), and the imbalanced expression in which the B genes had higher expression than the A genes in FJ fruits played important roles (Figure 7).

The ASE genes related to starch degradation and highly expressed during the FJ banana fruit ripening were screened, including four alleles: *MaAMY1*/*MbAMY3*, *MaBMY1*/*MbBMY2*, *MaBMY7*/*MbBMY8* and *MaDPE2*/*MbDPE2*. These typical genes were chosen for further RT-qPCR analysis (Figure 7). The results show that *MaAMY1*/*MbAMY3* exhibits imbalanced expression in FJ fruit with specific expression of the B gene, and the expression of *MaAMY1*/*MbAMY3* alleles in FJ banana fruit is much higher than in BX banana fruit. *MaBMY1*/*MbBMY2* and *MaBMY7*/*MbBMY8* exhibit imbalanced expression during the ripening process of FJ banana fruit, with these two alleles showing specific expression of the A gene. *MaBMY1*/*MbBMY2* is highly expressed during the turning stage. *MaDPE2*/*MbDPE2* is highly expressed in both FJ and BX banana fruits, with a higher expression at the turning stage. The expression level in BX banana fruit is slightly but significantly higher than in FJ banana fruit. It is speculated that *MaAMY1*/*MbAMY3* may be a key gene for starch degradation in banana fruit. Spearman correlation analysis revealed the associations between the starch degradation ASE genes (*AMY*/*BMY*/*DPE*) expression and starch content during postharvest ripening of FJ bananas, with the association of the relevant A genes in BX bananas as reference. In BX bananas, all the selected genes showed negative correlation with the starch contents (Supplementary Figure S4D), indicating these genes are involved in starch degradation. In the FJ, except *MbAMY3,* all the selected genes showed negative correlation (Supplementary Figure S4C). *MbAMY3* had higher expression than the allelic A gene in FJ and the gene in BX fruits, while the other three selected B genes in FJ showed lower expression than the allelic A gene (*MaBMY1*/*MbBMY2* and *MaBMY7*/*MbBMY8)* or lower expression of the gene in BX fruits (*MaDPE2*/*MbDPE2*) (Figure 7). The differentiated expression of these allelic genes is correlated to the similar starch degradation pattern between FJ and BX bananas (Figure 6E).

## 3. Discussion

The regulation of hormones and transcription factors during banana fruit ripening and quality development has been intensively studied. However, the formation and molecular mechanism of ripening features in heterozygous banana fruit is largely unknown. In this study, we explored a global view of ASE genes through transcriptome analysis, aiming to address whether the specific expression of ASE genes was associated with postharvest characteristics in different varieties (BX and FJ) during the ripening stages. Potential key ASE genes involved in ethylene biosynthesis, starch degradation and cell wall degradation that are related to fruit ripening traits were identified.

An increasing number of studies indicated that ASE is common in plants containing two or more sets of homologous genes. In this study, we examined the allelic gene expression in BX and FJ banana fruits during the ripening stage by using a published RNA-Seq dataset and high-quality reference A- and B-genomes [22]. A large number of ASE genes associated with post-ripening traits were identified in the FJ (ABB group) fruit. We considered 2-fold expression ratios (B > 4A or B < A) as indicating strict allelic expression imbalances. Based on transcriptome analysis, 15,415 alleles were screened as DEG allele pairs involved in banana ripening from a total of 25,716 allele pairs. Among them, 27.2% (3 d) and 22.2% (6 d) of alleles from the 15,415 DEG allele pairs showed significant ASE in FJ fruit (Figure 1). In addition, the B genes showed an overall dominant expression in these allele pairs. The ASE phenomenon often occurs when one parental allele is advantageous while the other is not. Hybrids may express the correct allele at a higher level in response to the environment or during development [23]. ASE appears to be a common phenomenon in heterozygotes. In maize hybrids, approximately more than 14% of expressed genes exhibited allelic imbalanced expression [24]. Moreover, the researchers of a previous study found that 31% of analyzed genes showed ASE between the parental inbred lines in Arabidopsis seedlings [6]. Furthermore, in rice shoots, 24% of expressed genes exhibited differential expression [25]. In apple, transcriptome mapping of 13 developmental stages of the fruit revealed that 19% of the genes displaying ASE, affecting quality-related pathways [26], and recent studies have identified 2091 ASE genes using RNA-seq data from the cultivar ‘Royal Gala’, highlighted the role of ASE in regulating key traits such as flower color. The ASE of the *MdMYB110a* and *MdMYB10* genes plays a significant role in anthocyanin synthesis, affecting flower color [11]. Our previous studies identified ASE of *SGR1* in heterozygous banana hybrids, we compared chlorophyll degradation of four banana cultivars (Cavendish [AAA, BX], Mysore [AAB], Pisang Awak [ABB, FJ], and Bluggoe [ABB]), in the AAB/ABB banana cultivars, an imbalanced expression, in which the B-*SGR1* alleles maintained high expression while the A-*SGR1* was reduced by the high temperature, prevents high-temperature-induced green ripening. In contrast, the A-*SGR1* was reduced by the high temperature in AAA Cavendish bananas, leading to the green remaining under heat stress. This study provides the first evidence of ASE in banana hybrids, revealing ASE may exist widely among A and B subgenome alleles in polyploid cultivars [16]. The study of other research group using integrated multi-omics analysis also demonstrated predominant B-subgenome expression bias in triploid Pisang Awak. These findings suggest that allele dominance has an important impact on fruit ripening and starch metabolism [22]. Here, we present the first systematic investigation of ASE dynamics during postharvest ripening in FJ banana, revealing genome-wide imbalanced expression patterns between A and B subgenomes and their impact on fruit ripening and metabolism. Moreover, this study identified and characterized DEGs related to ethylene biosynthesis, starch degradation and cell wall degradation. Among the DEGs identified, 62.5–83.3% exhibited ASE in the ethylene biosynthesis pathway (Figure 3 and Table 1); 50–75% of *AMYs*, 12.5 of *BMYs* and 0–50% of *DPEs* related to starch degradation exhibited unequal allele expression (Figure 4 and Table 2) and 34.6–90.9% showed ASE in the cell wall degradation pathway (Figure 5 and Table 3). A large number of ASE genes may be the main reason for the variety of differences in bananas, and these ASE genes are widely enriched in the basic biological function pathways associated with fruit ripening.

FJ banana cultivars exhibit shorter shelf lives compared to other commercial varieties [27]. Previous reports have indicated that ethylene plays a key role in the regulation of banana fruit ripening [28]. The core enzymatic steps in the ethylene biosynthesis pathways include SAMS, ACS and ACO [22]. The two key enzymes ACS and ACO regulate the production of ethylene, serving as the rate-limiting factors [29]. Ethylene biosynthesis genes, including *MaACS1*, *MaACO1*, *MaACO4*, *MaACO5* and *MaACO8*, were found to be upregulated in ripening bananas [15]. In this study, the results indicate that the ripening of FJ fruit is faster than that of BX fruit in terms of color, fruit softening, starch degradation and ethylene peak. More ethylene was released in the FJ fruit than in the BX fruit during the whole ripening process (Figure 6). Potential key genes of ASE involved in ethylene biosynthesis were screened. Five upregulated ethylene-biosynthesis-related alleles were detected in both FJ and BX banana fruits. *MaACO2*/*MbACO6*, *MaACO3*/*MbACO7*, *MaACO8*/*MbACO13*, *MaACO6*/*MbACO17* and *MaACS7*/*MbACS7* alleles may play an important role in ethylene biosynthesis, and the RT-qPCR analysis further showed that the expression of the B genes of all of these key enzyme genes in banana is more active than that of the A genes. The expression levels of *MbACO6*, *MbACO7* and *MbACO13* in FJ banana fruit were markedly higher than those of *MaACO2*, *MaACO3* and *MaACO8* in both FJ and BX banana fruits. The B genes of *MbACO6*, *MbACO7* and *MbACO13* may be the key genes for FJ banana fruit ripening (Figure 7). ASE may have an important effect on ethylene biosynthesis in postharvest banana fruit ripening, causing FJ (ABB) fruit to ripen faster than BX (AAA) fruit.

Bananas undergo substantial starch accumulation during growth, which is transformed into sugars during postharvest maturation [30,31]. During the ripening process of bananas, the metabolism of starch-to-sugar contributes to the softening and sweetness development of banana fruit pulp, and the degradation of starch into sugars leads to a rapid decrease in starch content and fruit firmness, as well as an increase in sweetness [31,32]. The key genes involved in regulating starch-to-sugar conversion during banana ripening include *AMYs*, *BMYs* and *DPEs* [19,20]. Banana fruit pulp accumulates substantial starch reserves, constituting 20–25% of the fresh weight at harvest maturity [20]. Previous reports suggest that the starch content in FJ fruit is higher than that in BX fruit [33]. In this study, the starch content was 22.36% for FJ fruit and 18.94% for BX fruit at the 0 d harvesting stage (Figure 6). The starch degradation processes and associated products vary among FJ and BX fruits; the decline in starch content in FJ fruit is faster than that in BX fruit. Four upregulated starch-degradation-related genes were detected in both FJ and BX banana fruits. The ASE alleles of *MaAMY1*/*MbAMY3*, *MaBMY1*/*MbBMY2*, *MaBMY7*/*MbBMY8* and *MaDPE2*/*MbDPE2* may play an important role in the degradation of starch. During fruit ripening, these genes exhibited significant upregulation, correlating with the progressive decline in starch content. *MaAMY1*/*MbAMY3* exhibited high expression during fruit ripening, which may be a key gene for starch degradation in banana fruit (Figure 5 and Figure 7). The specific expression of *MbAMY3* in FJ may be the reason for the faster degradation of starch in FJ than in BX. To date, *AMY* and *BMY* family genes have been reported to contribute to starch degradation during banana ripening [22], and the activities of α-amylases and β-amylases associated with starch degradation were confirmed in ripening bananas [19,20,34]. AMY genes appear to have a significant function in starch degradation; during banana ripening, the increase in AMY activity is strongly associated with reduced starch levels [31]. The *BMY* gene regulates the synthesis of β-amylases, producing high-molecular-weight dextrin from linear starch or pectin; β-amylases are crucial in the starch degradation process [35]. *MaBMY1*/*MbBMY2* and *MaBMY7*/*MbBMY8* play important roles in regulating the degradation of starch in FJ and BX fruits, causing differences in degradation products in banana fruits (Figure 5 and Figure 7). Overall, the results show that ASE is commonly present in FJ banana fruit during ripening. ASE genes may lead to differences in ripening traits in hybrid varieties and can provide new targets for molecular breeding. The functional differences in alleles are also worth further exploration.

## 4. Materials and Methods

### 4.1. Plant Material and Treatments

The fruits of two banana cultivars, BX (*Musa*, AAA, Cavendish subgroup, cv. Baxi) and FJ (*Musa*, ABB, Pisang Awak subgroup, cv. Fenjiao), at around 80% maturation were harvested from an orchard in Guangzhou, China. Banana fruits with a uniform shape, with sufficient maturity and without visual defects were chosen for the experiments. Surface sterilization was performed by first treating the fruits with 1% hypochlorite solution for one minute, then transferring them to a 0.05% Sporgon (*w*/*v*; Aventis, Madrid, Spain) solution for three minutes. Afterwards, the fruits were exposed to 100 μL L^−1^ ethylene for 24 h to initiate ripening, and then stored at 20 °C and 90% relative humidity for 6 days. The fresh tissue was sampled at 0−6 days until the fruit ripened completely. This study used 108 BX banana fruits and 108 FJ banana fruits. For daily photographic monitoring and *h°* value, 3 fruits, each as a replicate, were systematically positioned under standardized imaging conditions. Three biological replicates were picked from each banana cultivars daily and subjected to the measurements: three fruits for firmness testing with each as a replicate, nine fruits for ethylene production measurement with three fruits for each replicate, and three fruits for tissue sampling (starch content and RNA extraction) with each for a replicate. The samples were quickly frozen using liquid nitrogen and subsequently maintained at −80 °C.

### 4.2. Assessment of Fruit Ripening Parameters

The color of the banana peel was detected with a Minolta chroma meter (model CR-300, Konica Minolta, Osaka, Japan), and the results were presented as *h°*. The firmness of fruit was monitored with a penetrometer (no.5542; Instron Co., Norwood, MA, USA) that had a 6 mm diameter cylindrical probe. Ethylene production was determined according to Ref. [36], and measured by a gas chromatograph (GC-2014, Shimadzu Co., Kyoto, Japan). The total starch content was measured using a commercial Starch Assay Kit (Redshineen Co., Ltd., Guangzhou, China).

### 4.3. Transcriptome and Allele-Specific Expression Analysis

The transcriptome data have been made publicly available in the CNSA database (CNP0000292) [22]. Fruit pulp samples from BX (0/8/14 DPH) and FJ (0/3/6 DPH) cultivars were subjected to transcriptome sequencing. The raw reads were refined into clean reads by eliminating low-quality sequences, mismatches and adaptor sequences (Supplementary Table S1). Transcriptome assembly was performed using Cufflinks after mapping to A/B genome references, with RPKM-normalized expression profiling. The DESeq package was applied to screen for differentially expressed genes (DEGs). Those allelic genes of FJ banana with a fold-change of ≥2 and a false discovery rate (FDR) < 0.05 were considered significant DEGs. Allele-specific expression (ASE) was identified when the allelic expression fold difference between the two alleles (A and B) was as follows: B > 4A or B < A. In normal diploid heterozygous loci, the 2-fold threshold has become the standard in both plant and animal ASE studies, such as maize [4], barley [9], and rice [37]. As the genotype of FJ banana belongs to the ABB group (containing two B-genomes and an A-genome), we decided the 2-fold threshold in FJ should be adjusted to 4 folds of B to A or B less than A, namely B > 4A or B < A, as the identification of ASE between the two alleles (A-, B-).

Gene ontology (GO) term annotations were derived from BLAST results using Blast2Go tool (v6.0) to analyze ASE genes [38]. The Kyoto Encyclopedia of Genes and Genomes (KEGG) database was used to analyze the pathway annotation of ASE genes (http://www.genome.jp/kegg/) (accessed on 4 December 2024). A heatmap of related ASE genes was generated with the “pheatmap” package (R version 4.1.0).

### 4.4. Allele-Specific qRT-PCR Analysis

Total RNA was isolated from fresh of FJ and BX banana after ethylene treatment at 3 ripening time points. For BX bananas, samples were collected at 0, 3 and 5 DPH, while FJ bananas were sampled at 0, 2 and 4 DPH, using the hot borate method [39]. Then, the RNA samples underwent deoxyribonuclease I digestion treatment with the RNAse-free kit (Takara, Code No. 2212, Kusatsu, Japan), and cDNA synthesis was performed using PrimeScript™ RT reagent Kit with gDNA Eraser (Takara, Code No. RR047A, Kusatsu, Japan). Allele-specific primers were designed to distinguish the A- and B- gene alleles at the specific polymorphic code using Primer3 software (http://primer3.ut.ee/) (accessed on 3 June 2024). The expression of ASE genes was normalized using MaRPS4 [40]. All PCR primers are detailed in Supplementary Table S2. The RT-qPCR assays were conducted following the method we previously proposed [16] with the 2^−ΔCt^ calculation approach [41].

### 4.5. Statistical Analysis

The experiments followed a randomized design, and three biological replicates were used for different cultivars. ASEs from the DEGs were identified using the fold-change threshold (B > 4A or B < A) and FDR-adjusted *p*-value < 0.05. Differences in the fruit ripening parameters were analyzed through one-way ANOVA, the least significant difference (LSD) at the 5% level was determined through Student’s *t*-tests (SPSS 20.0, Chicago, IL, USA).

## 5. Conclusions

This work presents the allele-specific expression gene profiles related to banana fruit ripening for the first time, including ethylene biosynthesis, starch degradation and cell wall material degradation. ASE genes associated with post-ripening traits were identified in FJ (ABB group) fruit through comparison with the *Musa acuminata* and *Musa barbarian* reference genomes. The candidate key ASE genes controlling the correlation differences in fruit ripening were screened. The alleles *MaACO2*/*MbACO6*, *MaACO3*/*MbACO7*, *MaACO8*/*MbACO13*, *MaACO6*/*MbACO17* and *MaACS7*/*MbACS7* have been identified as crucial for ethylene biosynthesis, while *MaAMY1*/*MbAMY3*, *MaBMY1*/*MbBMY2*, *MaBMY7*/*MbBMY8* and *MaDPE2*/*MbDPE2* alleles may play an important role in the degradation of starch. This study adds to the literature on the exploration of core molecular mechanisms underlying postharvest banana fruit ripening quality and contributes to the molecular breeding of bananas.

## Figures and Tables

**Table 1:** The number of DEGs involved in the ethylene biosynthesis pathway that exhibit ASE during FJ fruit ripening at a minimum allele fold difference of 2.0×.

Gene Name	Number of DEGs	A > B	B > 4A	ASE Ratio (%)
SAMS-3d	6	5	0	83.33
SAMS-6d	6	5	0	83.33
ACS-3d	3	0	2	66.67
ACS-6d	3	0	2	66.67
ACO-3d	8	2	3	62.50
ACO-6d	8	1	4	62.50

**Table 2:** The number of DEGs associated with the starch degradation pathway that exhibit ASE during FJ fruit ripening.

Gene Name	Number of DEGs	A > B	B > 4A	ASE Ratio (%)
AMY-3d	4	0	2	50
AMY-6d	4	1	2	75
BMY-3d	8	0	1	12.5
BMY-6d	8	0	1	12.5
DPE-3d	2	0	0	0
DPE-6d	2	1	0	50

**Table 3:** The number of DEGs involved in the cell wall degradation pathway that exhibit ASE during FJ fruit ripening.

Gene Name	Number of DEGs	A > B	B > 4A	ASE Ratio (%)
EXPANSIN-3d	26	5	4	34.62
EXPANSIN-6d	26	6	8	53.85
XTH-3d	34	12	14	76.47
XTH-6d	34	7	12	55.88
PL-3d	11	7	3	90.91
PL-6d	11	3	2	45.45
PE-3d	26	7	9	61.54
PE-6d	26	9	5	53.85
PG-3d	22	5	4	40.91
PG-6d	22	4	8	54.55

## Data Availability

The original contributions presented in this study are included in the article/Supplementary Material. Further inquiries can be directed to the corresponding authors.

## Supplementary Materials

The following supporting information can be downloaded at: https://www.mdpi.com/article/10.3390/ijms26094090/s1.
