# Supplementary material for: Impact of Allele-Specific Expression on Ripening and Quality Characteristics of ABB Banana Fruit
Source: Int J Mol Sci. 2025 Apr 25;26(9):4090. doi: 10.3390/ijms26094090 (PMC12071630; doi:10.3390/ijms26094090)
Supplement: Supplementary file 1 [file ijms-26-04090-s001.zip › ijms-3549329-supplementary.pdf]

Sample	RawDatas	CleanData(%)	Adapter(%)	LowQuality(%)
BX-0d-1	7554406	7516294 (99.50%)	5620 (0.07%)	32492 (0.43%)
BX-0d-2	7456670	7400756 (99.25%)	5454 (0.07%)	50460 (0.68%)
BX-8d-1	7497756	7453616 (99.41%)	3994 (0.05%)	40146 (0.54%)
BX-8d-2	7425118	7368616 (99 24%)	4096 (0.06%)	52406 (0.71%)
BX-14d-1	7490418	7447382 (99.43%)	4356 (0.06%)	38680 (0.52%)
BX-14d-2	7457540	7406898 (99.32%)	4066 (0.05%)	46576 (0.62%)
FJ-0d-1	7436522	7387712(99.34%)	3944 (0.05%)	44866 (0.60%)
FJ-0d-2	7487784	7438740(99.35%)	3958 (0.05%)	45086 (0.60%)
FJ-3d-1	7465278	7412702(99.30%)	4232 (0.06%)	48344 (0.65%)
FJ-3d-2	7435538	7381904 (99.28%)	4516 (0.06%)	49118 (0.66%)
FJ-6d-1	7236864	7188196 (99.33%)	3968 (0.05%)	44700 (0.62%)
FJ-6d-2	7441514	7387534 (99.27%)	3882 (0.05%)	50098 (0.67%)

Supplementary Table S1. Summary information of sequencing data and mapped ratios of BX and FJ fruit during postharvest ripening.

Gene	Gene ID	Forward primer (5' to 3')	Reverse primer (5' to 3')
MaACS7	Ma04_g35640	GGGGTCATCCAAATGGGACTC	GGTGCAGATCGAAGCCTGC
MbACS7	Mb04t36040	GGGTGTCATCCAAATGGGACTT	GGTGCAGATCGAAGCCTGT
MaACO2	Ma05_g09360	TCAAGCACGGAAGACGAC	CAAATGTCTTCTTCAGATGATCATC
MbACO6	Mb05t08270	TTCACCAAGTGTGCAGGAAA	CCGAGTCGTAGAACCTTTCCTTC
MaACO3	Ma06_g02600	GGAGAGCTTCTACGAGTCGGA	GGGGAGATCATTGATGTTGGG
MbACO7	Mb06t02410	GGAGAGCTTCTACGAGTCGGG	GGGGAGATCATTGATGTTGGA
MaACO6	Ma06_g14420	ATTTCTCGAAGTTGGAGGGGAAGG	ACGCGTTCCAAGAGTTCCACT
MbACO17	Mb00t00190	ATTTCTCAAAGTTGAAGGGCAAGC	CGATCCAGAAGCTCCACC
MaACO8	Ma07_g19730	GAAGGCGATGAAGGAATTTGCG	CCAAAGGTTGGCCCCTTGG
MbACO13	Mb07t09090	AGGAATGCGATGAAGGAATTTGCT	CAAAGGTTGGCCCCTTGC
MaAMY1	Ma03_g07140	AAGATCTACGTCGAACAGACGCAT	TTCTGATCGTATGCTAGCTTCCCA
MbAMY3	Mb03t07080	AAGATCTACGTCGAACAAACGCAG	TGATCGTATGCTGGCTTCCCG
MaBMY1	Ma01_g10710	TGGACGTGAAGGAAGAGGATGAC	TGATCCCGATCCCGATCCCG
MbBMY2	Mb01t20290	TCGGCAGCAGCACTGACTCT	GGAAGCATAACATAAACCGGGACG
MaBMY7	Ma05_g07800	TAGGGTTCGGGAACTCGGTCA	AATCGACCGCTTCCTCCATGCC
MbBMY8	Mb05t09700	AGGCAGGATTGATGGTGAAACAA	TCGAACCCGATCCTGCTGATC
MaDPE2	Ma03_g08680	CAGGAAGCTTCGGAAACTCTTTTCG	CCAATTCCTTAGAACTTTCATCGGC
MbDPE2	Mb03t08550	GGAAGCTTCGGAAGCTCTTTTCT	CCAATTCCTTAGAACTTTCATCAGT
MaRPS4	HQ853247.1	TGAGAGTGGCTTGACCCTGA	GTGACATTTAGTCGTCTGCTGG

Supplementary Table S2. Gene ID and primers for quantitative real-time PCR analysis.

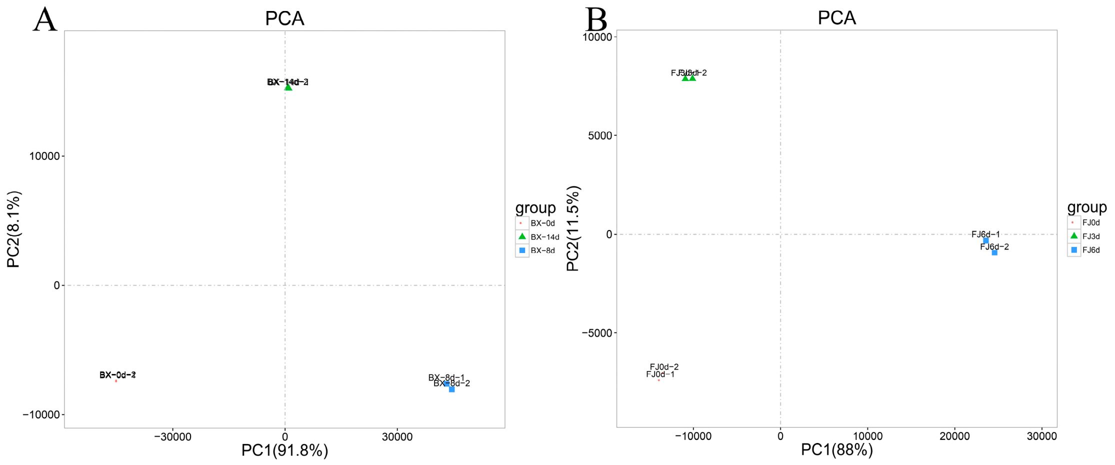

Supplementary Figure S1. Principal component analysis (PCA) of samples of BX and FJ banana fruit at different ripening stages.

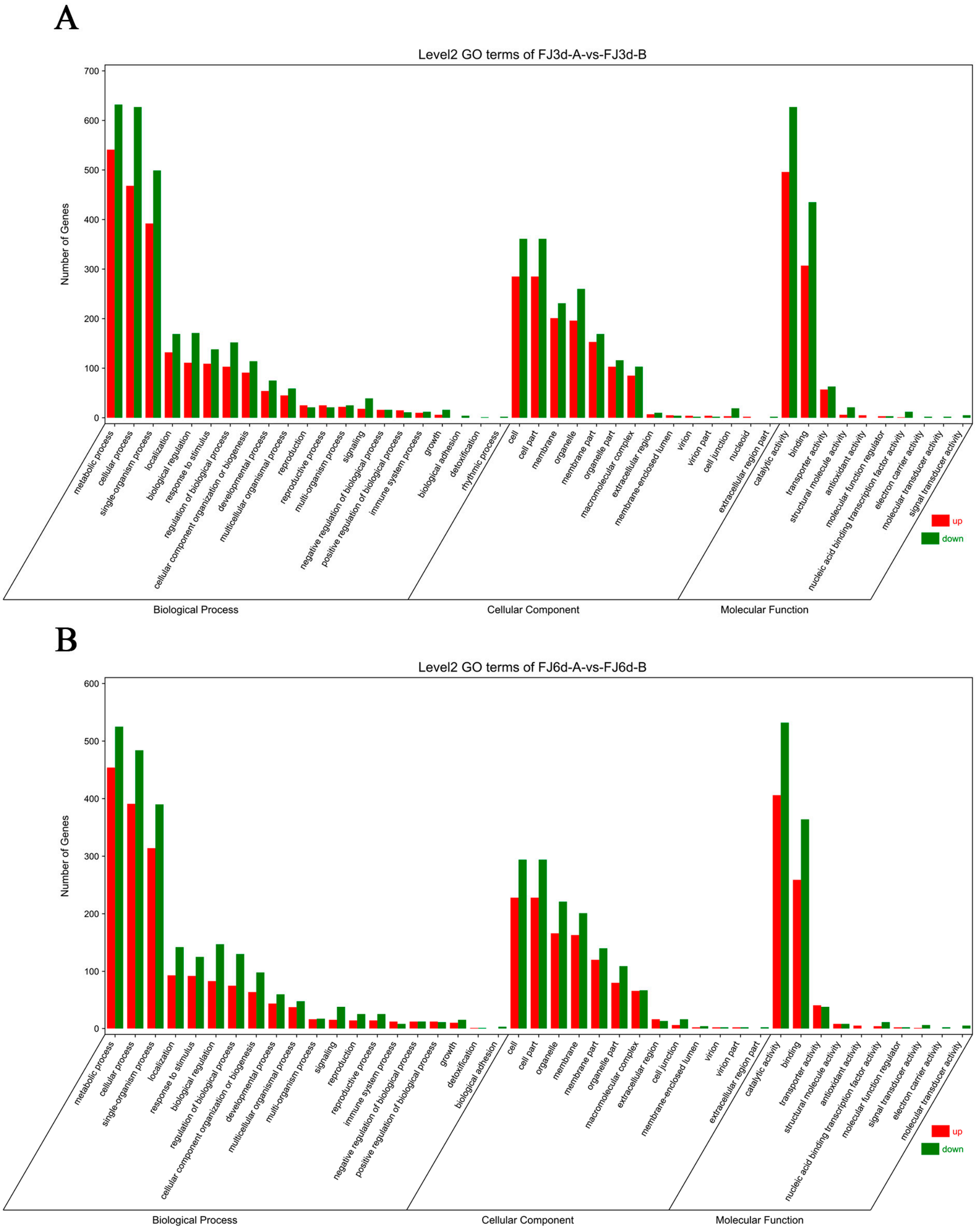

Supplementary Figure S2. The GO term classifications of DEGs displaying ASE in FJ bananas during the fruit-ripening process. The y-axis indicates the number of annotated unigenes.

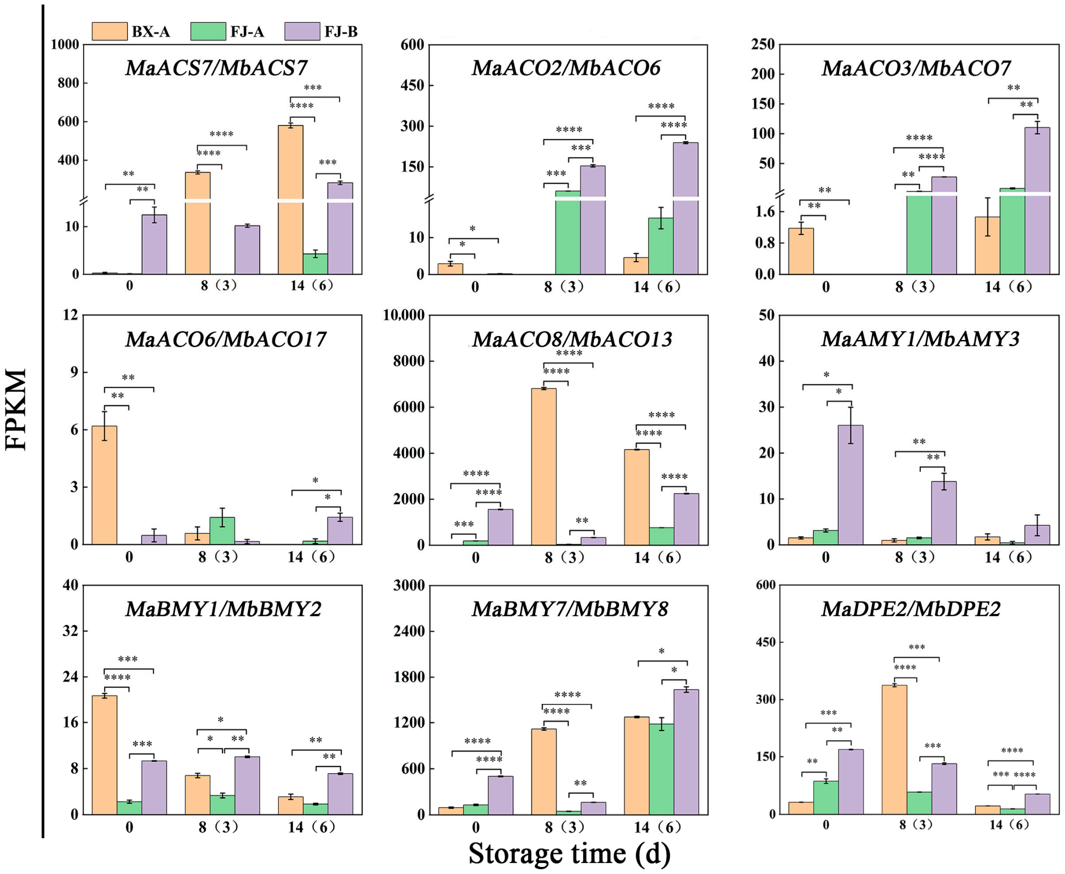

Supplementary Figure S3. Relative expression levels of selected ASE genes in BX and FJ fruits at three time points during storage detected by RNA-seq. Each data point represents the average ± standard error (SE) derived from two independent biological replicates. The Y-axis represents the FPKM level of each gene.

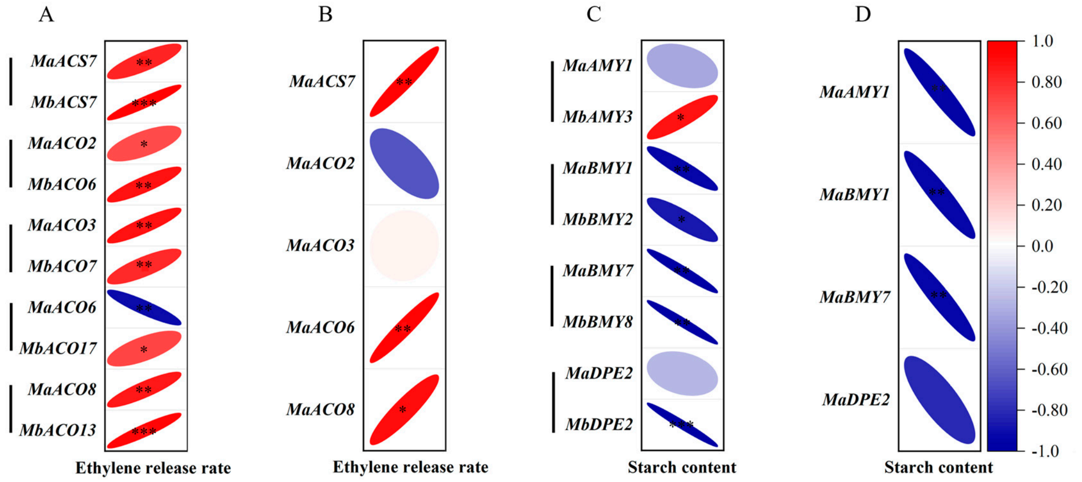

Supplementary Figure S4. Correlation of the ethylene/starch contents and the expression patterns of the ethylene biosynthesis-related and starch degradation-related ASE genes during the fruit ripening of FJ and BX. Correlation of gene expressions of ASE genes (*ACS*/*ACO*) with ethylene release rates during FJ (A, the A/B-genes) and BX (B, the A-genes) banana ripening. Correlation analysis gene expressions of ASE genes (*AMY*/*BMY*/*DPE*) with starch content during FJ (C, the A/B-genes) and BX (D, the A-genes) banana ripening. The allelic genes from A or B genome in FJ were marked by the left lines of the gene names in (A) and (C). Values are the mean ± standard error (n=3). Asterisks in the ellipse represent significant correlation (Significance levels: $*p \leq 0.05$, $**p \leq 0.01$, $***p \leq 0.001$)
